# Check point to get adequate weight loss within 6-months after laparoscopic sleeve gastrectomy for morbid obesity in Asian population

**DOI:** 10.1038/s41598-020-69714-4

**Published:** 2020-07-30

**Authors:** Chung-Yen Chen, Cheng-Hung Lee, Hui-Ming Lee, Wen-Yao Yin, Wei-Leng Chin, Ming-Hsien Lee, Jian-Han Chen

**Affiliations:** 1 Bariatric and Metabolism International Surgery Center, E-Da Hospital, Kaohsiung, Taiwan; 2 Division of General Surgery, E-Da Hospital, Kaohsiung, Taiwan; 3 Department of Family Medicine, E-Da Hospital, Kaohsiung, Taiwan; 4 Division of General Surgery, E-Da Cancer Hospital, Kaohsiung, Taiwan; 5 School of Medicine, College of Medicine, I-Shou University, Kaohsiung, Taiwan; 6 Department of General Surgery, Dalin Tzu Chi Hospital, Buddhist Tzu Chi Medical Foundation, Chia-Yi, Taiwan; 7 Division of Metabolic and Bariatric Surgery, Taichung Tzu Chi Hospital, Buddhist Tzu Chi Medical Foundation, Taichung, Taiwan; 8 School of Medicine, Tzu Chi University, Hualien, Taiwan

**Keywords:** Risk factors, Obesity

## Abstract

Purpose of this study is to develope a scoring system to predict the likelihood of excess body weight loss (EBWL) ≥ 50% 6-months after laparoscopic sleeve gastrectomy (LSG). From April 2016 to September 2018, data was collected from 160 patients (BMI ≥ 32) who underwent primary LSG with at least 6-months follow-up. They were separated into score generation (operated by one surgeon, n = 122) and validation groups (operated by 3 different surgeons, n = 38). EBWL at 6-months ≥ 50% was considered adequate weight loss. Independent variables including age, gender, initial body mass index (BMI), comorbidities, life-style habits, percentage of EBWL and percentage of total body weight loss at 1-week, 1-month, and 3-months were analyzed with mutivariate logistic regression to generate the scoring system. The system was applied to internal and external validation groups to determine efficacy. As results, between the score generation and internal validation groups, the only significant difference in patient characteristics was in exercise participation. EBWL at 1-month > 19.5% (1 point) and EBWL at 3-months > 37.7% (2 points) were identified as independent factors to predict EBWL at 6-months ≥ 50%. When scores were > 1, the system had 94.03% positive predictive value (PPV) and 81.82% negative predictive value (NPV) (AUC: 0.923). Internal validation scores > 1 had a 95.83% PPV and 85.71% NPV (AUC: 0.975). External validation results showed 88.59% PPV and 72.00% NPV (AUC: 0.802). We concluded that this scoring system provides a reliable, objective prediction of EBWL at 6-months ≥ 50%. Patients requiring more aggressive clinical follow-up and intervention can be detected as early as 1- to 3-months after LSG.

## Introduction

As the prevalence of morbid obesity increases worldwide, bariatric surgery is becoming increasingly common. At the onset, it is very important to identify the 10–20% of patients who are at-risk for inadequate weight loss after surgery^1–3^. Poor postoperative weight loss may also influence the metabolic effect of bariatric surgery, limiting the expected health benefits such as improvement in diabetes management^4,5^, hypertension remission, and resolution of dyslipidemia, sleep apnea and multiple other comorbidities^5^.

In addition to exploring risk factors, several published models are designed to predict adequate weight loss after bariatric surgery. For Roux-en-Y gastric bypass (RYGB), Moret al.^6^ found that a quartile attribution for EWL at the 1-month postoperative visit was maintained throughout the first year with 39% PPV and 81% NPV. Al-Khyattet al.^2^ established a statistically significant model, with predicted %EWL = constant − (9.2 * BMI) − (2.9 * age) + (4.8 * preoperative EWL) − (3.1 * TtS) − (6.2 * DM), and a correlation coefficient of 0.43. The model predicted EWL at 12-months in 43% of their data. Slotman^7^ published another model to predict weight and co-morbidities at 2-, 6-, 12-, 18-, and 24-months via preoperative data. These models were generated from gastric bypass patients. There are no validation studies applying these models to LSG.

Early postoperative weight loss is reported to be an important predictive factor for overall weight loss 1- and 3-years after bariatric surgery non-Asian^5,8,9^ and Asian populations^10^. Kanerva et al.^11^ demonstrated that in the short-term, considered to be 6-months after bariatric surgery, changing dietary macronutrient composition affects 10-year postoperative weight. Therefore, early postoperative detection of poor weight loss is key to clinical decision-making regarding treatment interventions. Moreover, Manning et al.^8^ reported weight loss velocity between postoperative 3- to 6-months is an independent predictor for a maximal percentage of weight loss in LSG. Their study showed that weight loss percentage is a stronger predictor in LSG patients compared to RYGB patients. We believe that predictive models generated from RYGB may not fully apply to LSG.

There are weight loss predictor models generated from LSG. Cottam et al.^9^ established a prediction model based on comorbidities, including diabetes and/or sleep apnea, and the %EWL at 1-and 3-months after LSG to predict %EWL > 55% at 1 year with a 71% sensitivity, 91% specificity, 72% PPV and 91% NPV. Van de Laaret al.^3^ presented bariatric weight loss charts with standard deviation and percentile curves which aims to assess weight loss, weight-regain, and poor responders up to 7 years after sleeve gastrectomy and was validated by large studies (n > 500), reporting weight loss results after LSG with BMI > 35 kg/m^2^ and age ≥ 18 years with a minimum of 5-years follow-up. However, they were not validated for Asian populations.

This study aimed to generate a predictive scoring system to identify patients with more than 50% excess body weight loss (EBWL) 6-months after LSG.

## Methods

This study was fully evaluated and approved by the Institutional Review Board of Eda Hospital and was conducted in accordance with the principles of the Helsinki Declaration. The institutional ethics committee waived the need for patients’ written informed consent for this retrospective analysis of clinically acquired data.

From April 2016 to September 2018, data was collected from 334 consecutive adult patients who underwent laparoscopic sleeve gastrectomy (LSG) and follow-up for at least 6 months at a single-site. Patients with incomplete weight data (n = 150), BMI < 32 (n = 23) and revisional surgery (n = 1) were excluded from the analysis. In addition, data from 811 patients at two additional centers was used for external validation of the scoring method.

### Laparoscopic sleeve gastrectomy

The surgery protocol was the same for all patients at the single-site. Under general anesthesia and in a supine position, the abdomen was prepared aseptically with betadine as usual. A 36-Fr oral gastral (OG) tube was inserted orally as a guide during gastrectomy. One small skin incision on the umbilicus created a laparotomy for 15 mm trocar insertions. After setting up the laparoscopic instruments, the left lateral segment was elevated to expose the angle of His. Gastrolysis of the greater curvature of the stomach from the greater omentum was performed, and the tip of the OG tube remained along the lesser curvature as a guide for transection. Sleeve gastrectomy was performed from the antrum, 5 cm away from the pylorus, to the angle of His along the OG tube. Routine intraoperative endoscopic examination was performed. The resected portion of the stomach was removed and all wounds were closed in layers. All patients were hospitalized for 24–48 h of postoperative observation. Follow-ups occurred 1-week, 1-month, 3-months, 6-months and 1-year after LSG.

### Variables

Our primary endpoint was EBWL 6 months after LSG. Adequate weight loss 6 months post-surgery was defined as EBWL ≥ 50%. Parameters including age, sex, initial body mass index (BMI), comorbidities, lifestyle, and emotional status were evaluated. Patients’ emotional status was evaluated with the Taiwanese Depression Questionnaire and the Chinese Health Questionnaire, which have been found appropriate for use in patients undergoing bariatric surgery^12^. Diabetes was defined by antidiabetic medication or insulin use or a preoperative hemoglobin A1c level > 6.5%. Hypertension was defined by the use of anti-hypertensive medication or a preoperative blood pressure level of more than 140/90 mmHg.

Patients were divided into two groups based on the operating surgeon. One group was used to generate the predictive scoring (operated on by a single surgeon, n = 122) and the other group was used for internal validation of the scoring method (operated on by 3 different surgeons, n = 38). Ideal postoperative weight was defined as a BMI of 22. The percentage of EBWL (%EBWL) and percentage of total body weight loss (%TBWL) at 1-week, 1-month, 3-months, and 6-months were collected. After generating the scoring system, it was applied to the internal validation group to test the efficacy of the model. Finally, the scoring system was applied to the off-site patient data for external validation.

### Statistical analysis

SPSS software (IBM, Chicago, IL, USA) was used for the descriptive statistics and contingency tables. The patient characteristics and covariates were analyzed with Student’s *t *test, Mann–Whitney U-test, and chi-square or Fisher’s exact tests. A *p *value ≤ 0.05 was considered significant. All variables were analyzed with collinearity diagnostics to identify independent variables. Independent variables in the score generation group were analyzed by logistic regression to evaluate their ability to predict adequate weight loss. The cut-off values for continuous data were decided by the receiver operating characteristic (ROC) curve. A backward stepwise logistic regression model was applied for multivariate logistic regression. All variables with *p*-values less than 0.2 were inserted into the model. To generate a score, the regression coefficient was divided by 2 and rounded to the nearest whole number. Sensitivity, specificity, positive and negative predictive values, and ROC curves were used to evaluate the efficacy of the scoring system.

### Ethical approval

For this type of study formal consent is not required.

## Results

Table 1 summarizes the characteristics of the 160 patients included in the score generation (n = 122) and internal validation (n = 38) groups. There were no significant differences between the groups in age, sex, emotional status, comorbidities, and health habits, except for exercise participation (20.49% in the score generation group vs 39.47% in the validation group, *p* = 0.03).

**Table 1:** Clinical characteristics of the score generating and internal validation groups.

	Score generation group^α^		Validation group^α^		*p* value
	N = 122		N = 38		
**Age (y), mean (± SD)**	35.59	(9.51)	36.05	(10.61)	0.742
**Gender (no., %)**					0.195
Female	70	57.38	17	44.74	
Male	52	42.62	21	55.26	
**BMI** **Mean (± SD)**	40.68	(05.77)	39.42	(05.10)	0.257
**Mood status**					
TDQ (± SD)	15.45	(11.88)	14.79	(11.69)	0.710
CHQ (± SD)	4.70	(2.46)	4.39	(02.84)	0.487
**Comorbidity (no., %)**					
Diabetes mellitus	31	25.41	13	34.21	0.304
Hypertension	75	61.48	25	65.79	0.704
**Lifestyle (no., %)**					
Alcohol	12	9.84	9	23.68	0.050
Smoking	36	29.51	11	28.95	1.000
Betel nut	5	4.10	3	7.89	0.396
Sports	25	20.49	15	39.47	0.030*
**Postoperative results**					
EBWL 1-week	15.03%	(04.64%)	14.79%	(04.51%)	0.501
TBWL 1-week	6.51%	(01.67%)	6.39%	(01.87%)	0.344
EBWL 1-month	23.44%	(06.98%)	26.18%	(07.18%)	0.033*
TBWL 1-month	10.11%	(02.06%)	11.22%	(02.68%)	0.023*
EBWL 3-month	41.02%	(11.66%)	44.30%	(12.76%)	0.182
TBWL 3-month	17.75%	(03.71%)	19.06%	(05.00%)	0.083
EBWL 6-month	56.32%	(16.44%)	58.94%	(15.65%)	0.352
TBWL 6-month	24.42%	(05.59%)	25.38%	(06.20%)	0.310

*EBWL* excess body weight loss, *TBWL* total body weight loss.

*p < 0.05.

^α^All patients are Asian.

The internal validation group had a significantly higher average 1-month %EBWL and %TBWL compared to the score generation group (26.18% vs. 23.44% [*p* = 0.033] and 11.22% vs. 10.11% [*p* = 0.023], respectively). However, there were no significant differences between the groups at 6-months.

### Scoring

In the univariate analysis, preoperative BMI < 39.2, smoking habit, 1-week EBWL > 16.1%, 1-month EBWL > 19.5%, and 3-month EBWL > 37.7% were independent factors in detecting adequate weight loss 6 months after LSG (Table 2). After multivariate analysis, only 1-month EBWL (regression coefficient = 1.610, *p* = 0.014; Score: 1) and 3-month EBWL (regression coefficient = 3.871, *p* < 0.001; Score: 2) were independent factors. Thus, the 6M50LSG scoring system for predicting adequate weight loss 6-months after LSG was generated.

**Table 2:** The factors influencing adequate weight loss after laparoscopic sleeve gastrectomy.

	Univariant analysis		Multivariant analysis		B	Score
	OR	*p* value	Adjust OR	*p* value		
**Preoperative**						
Sex	00.648	0.246*				
Age ≤ 39.88	01.597	0.255*				
BMI ≤ 39.2	07.572	< 0.001*	02.194	0.249		
Diabetes mellitus	00.636	0.281*				
Hypertension	00.657	0.276*				
Alcohol	03.730	0.099*	00.310	0.342		
Smoking	02.609	0.030*	01.745	0.486		
Betel nut	00.000	0.999*				
Exercise	02.522	0.070*	00.365	0.356		
TDQ > 6	01.875	0.135*	03.989	0.064		
CHQ < = 4	01.616	0.197*	01.484	0.579		
**Postoperative**						
EBWL at 1 week > 16.1%	10.667	< 0.001*	01.647	0.571		
EBWL at 1 month > 19.5%	19.446	< 0.001*	04.851	0.017*	1.579	1
EBWL at 3 months > 37.7%	70.875	< 0.001*	48.166	< 0.001*	3.875	2

*B* regression coefficient, *CHQ* Chinese Health Questionnaire, *EBWL* excess body weight loss, *OR* odds ratio, *TDQ* Taiwanese Depression Questionnaire.

*p < 0.05.

The 6M50LSG scoring system was applied to the score generation group and evaluated by the ROC curve. When the score was > 1, it had 86.3% (95% CI 76.25–93.23%) sensitivity, 91.84% (95% CI 80.40–97.73%) specificity, 94.03% (95% CI 85.98–97.59%) positive predictive value (PPV), and 81.82% (95% CI 71.55–88.95%) negative predict value (NPV). The area under the curve (AUC) was 0.923 (*p* < 0.001).

### Internal validation

The 6M50LSG scoring system was similarly applied to the internal validation group. When the score was > 1, it had 92.00% (95% CI 73.97–99.02%) sensitivity, 92.31% (95% CI 63.97–99.81%) specificity, 95.83% (95% CI 77.71–99.35%) PPV, and 85.71% (95% CI 61.14–95.81%) NPV. The AUC was 0.975 (*p* < 0.001).

### External validation

The 6M50LSG scoring system was applied to the external validation group, which included 493 of 811 patients at two additional sites after applying inclusion criteria (Table 3). When the score was > 1, the model had 90.35% (95% CI 86.77–93.15%) sensitivity, 68.57% (95% CI 59.51–76.01%) specificity, 88.59% (95% CI 85.78–90.90%) PPV, and 72.00% (95% CI 64.77–78.25%) NPV. The AUC was 0.802 (*p* < 0.001).

**Table 3:** The AUC, sensitivity, specificity, positive predictive value (PPV) and negative predictive value (NPV) of the 6M50LSG scoring system in the score generator, internal validation, and external validation groups.

	Score > 1					
	Score generator		Internal validation		External validation	
Sensitivity	86.30%	(76.25–93.23%	92%	(73.97–99.02%)	90.53%	(86.77–93.15%)
Specificity	91.84%	(80.40–97.73%)	92.31%	(63.97–99.81%)	68.57%	(59.51–76.01%)
PPV	94.03%	(85.98–97.59%)	95.83%	(77.71–99.35%)	88.59%	(85.78–90.90%)
NPV	81.82%	(71.55–88.95%)	85.71%	(61.14–95.81%)	72.00%	(64.77–78.25%)
AUC	0.923		0.975		0.802	

*AUC* area under the curve, *NPV* negative predictive value, *PPV* positive predictive value.

### Long term followed up

**Figure 1:**
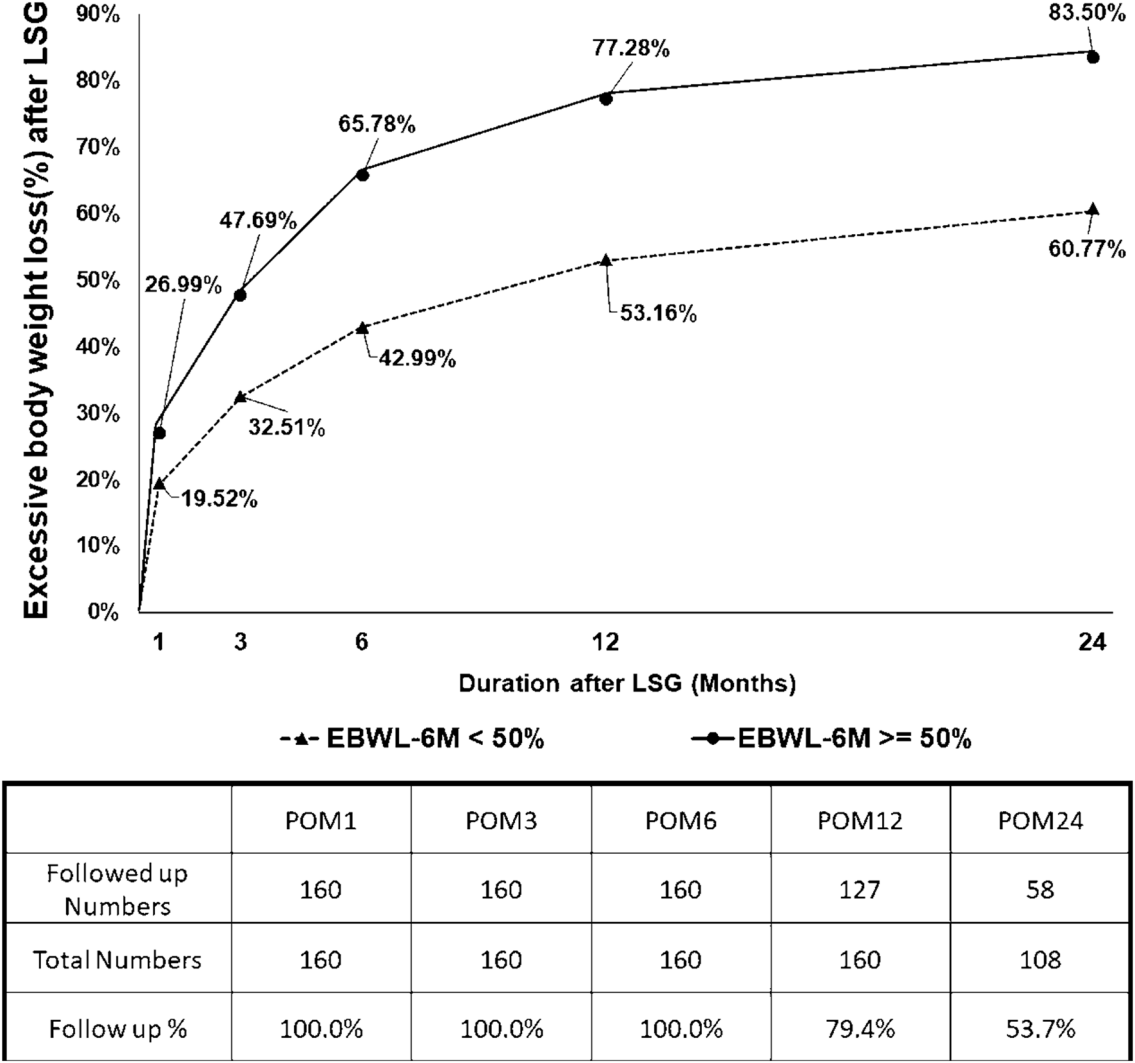
Excessive body weight loss after LSG.

The weight loss in the following period was listed in Fig. 1 and Table 4. The percentage of followed up in 1 year after LSG (POY1) is 79.4% and 53.7% in 2 years after LSG (POY2). Those patient with more than 50% EBWL 6-months after LSG had a significantly higher weight loss in POY 1 (Less 50% EBWL Group vs. more than 50% EBWL Group: EBWL 53.16% vs. 77.28%, *p* < 0.001) and POY 2 (Less 50% EBWL Group vs. more than 50% EBWL Group: EBWL 60.77% vs. 83.50%, *p* < 0.001). However, the weight difference between POY 1 and POY 2 is similar (Less 50% EBWL Group vs. more than 50% EBWL Group: Difference EBWL 2.36% vs. 2.18%, *p* = 0.945).

**Table 4:** Body weight loss after LSG.

	EBWL-6M < 50%		EBWL-6M ≥ 50%		*p*
	N = 62		N = 98		
**Postoperative body weight loss**					
EBWL% 1M	19.52%	(5.20%)	26.99%	(6.61%)	< 0.001*
TBWL% 1M	9.24%	(2.00%)	11.10%	(10.72%)	< 0.001*
EBWL% 3M	32.51%	(7.38%)	47.69%	(10.51%)	< 0.001*
TBWL% 3M	15.47%	(3.01%)	19.71%	(3.80%)	< 0.001*
EBWL% 6M	42.99%	(8.22%)	65.78%	(13.67%)	< 0.001*
TBWL% 6M	20.56%	(3.83%)	27.24%	(5.22%)	< 0.001*
EBWL% 1Y	53.16%	(13.37%)	77.28%	(16.19%)	< 0.001*
TBWL% 1Y	25.90%	(7.27%)	32.24%	(7.38%)	< 0.001*
EBWL% 2Y	60.77%	(18.51%)	83.50%	(17.47%)	< 0.001*
TBWL% 2Y	29.75%	(9.41%)	34.70%	(8.70%)	0.048*
Difference EBWL% POY2-POY1	2.36%	(7.45%)	2.18%	(10.35%)	0.945
Difference TBWL% POY2-POY1	1.30%	(3.81%)	1.15%	(4.04%)	0.899
Difference body weight POY2-POY1(Kg)	− 1.77	(5.06)	− 1.28	(4.04)	0.701

*BMI* body mass index, *EBWL* excess body weight loss.

*p < 0.05.

### Applying the scoring system proactively

**Figure 2:**
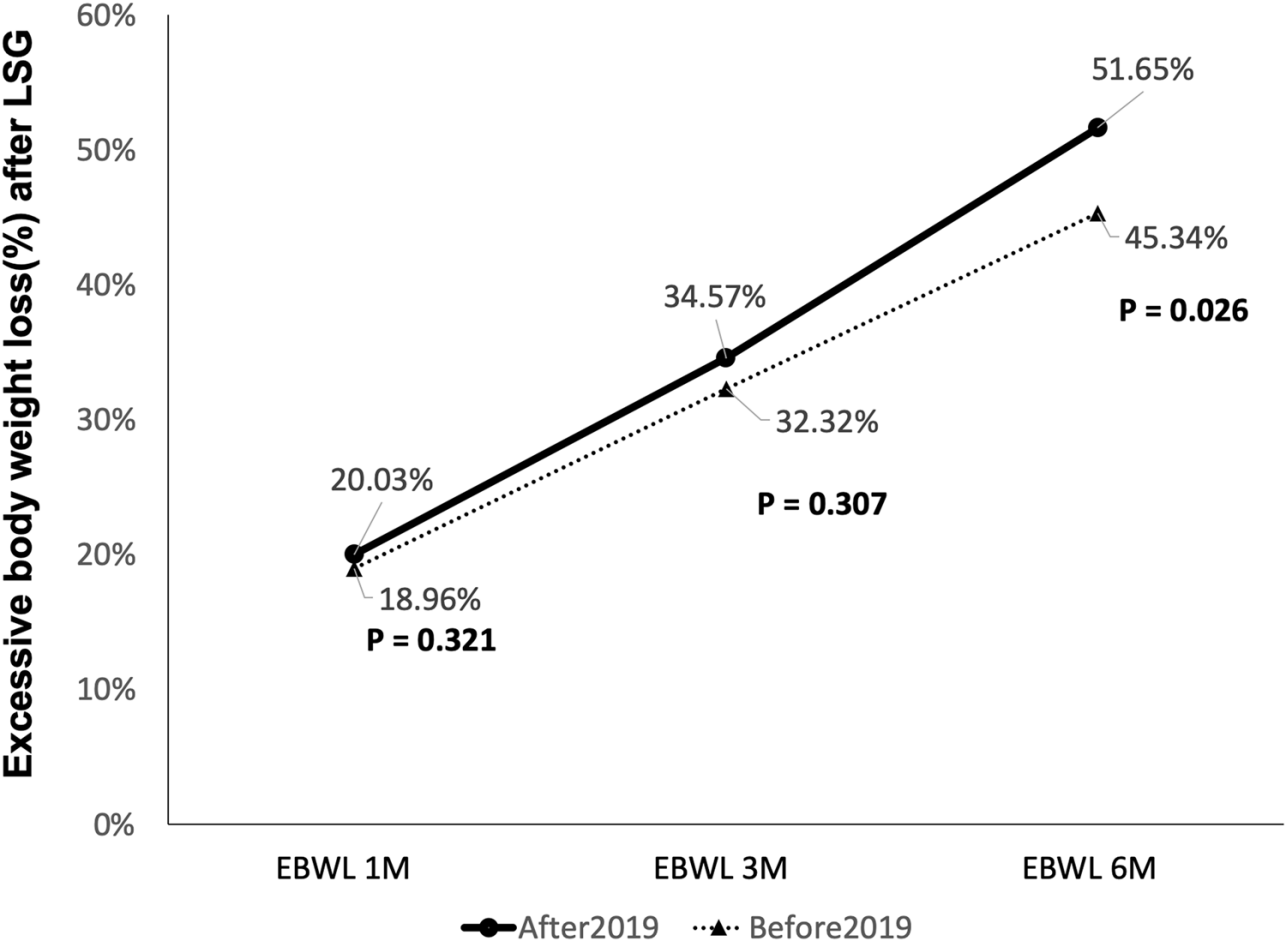
Excessive body weight of patients who did not meet the goal after LSG.

From 2019/01 to 2019/05, 28 consecutive patients underwent LSG in our institution. Eight of them did not meet postoperative weight loss goals, including 3 with 1 month EBWL ≤ 19.5% and 5 with 1 month EBWL > 19.5% but 3 month EBWL ≤ 37.7%. We increased the follow-up to monthly visits through postoperative month 6. Moreover, we asked these patients to complete photo food records and upload them via social media software to help dietitians correct their eating habits and food selection. Compared to previous patients who did not meet weight goals (Table 5), patients in 2019 had a higher rate of adequate weight loss but the difference was not significant. However, patients in 2019 had significantly higher 6 month EBWL than patients before 2019 (51.65% vs. 45.34%, *p* = 0.026) (Fig. 2).

**Table 5:** Clinical characteristics of patients who did not meet the goal after LSG.

	Before 2019		2019		*p *value
	N = 87		N = 8		
**Age (y), mean (± SD)**	36.61	(10.32)	32.64	(9.92)	0.300
**Gender (no., %)**					0.472
Female	55	63.22	4	50.00	
Male	32	36.78	4	50.00	
**BMI** **mean (± SD)**	43.60	(5.26)	49.29	(8.74)	0.007*
**Adequate weight loss**	21	24.13	3	37.50	0.412
**Postoperative results**					
EBWL 1-month	18.96%	(3.96%)	20.03%	(2.43%)	0.321
EBWL 3-month	32.32%	(6.02%)	34.57%	(4.77%)	0.307
EBWL 6-month	45.34%	(10.86%)	51.65%	(7.56%)	0.026*

*BMI* body mass index, *EBWL* excess body weight loss.

* *p* < 0.05.

## Discussion

In this study, we generated the 6M50LSG scoring system to predict adequate weight loss (≥ 50% EBWL) within 6 months after LSG. Results from the 3 data sets tested show that the 6M50LSG scoring system has outstanding discrimination.

In the literature, the target for adequate weight loss after LSG has been reported as 50% EBWL within 6-months^13^, 50% EBWL within 1-year^5^, 55% EBWL within 1-year^9^, 50% EBWL within 2-years^14,15^, and even 50% EBWL within 3-years^10^. Our results used a relatively shorter follow-up period in comparison. However, it was reported that the strength of association between maximum weight loss and %EBWL in the immediate postoperative period and the first 3-months after LSG is twice as strong as in RYGB^8^. Moreover, Kanerva et al.^11^ demonstrated that in the short-term, considered 3 months after bariatric surgery, changing dietary macronutrient composition was associated with 10-year weight change. Better weight loss at 10 years was seen with diets higher in the proportion of protein than fat, higher in carbohydrate than fat, and higher in protein than carbohydrates.

Reported predictors for poor postoperative weight loss have included older age^2,16,17^, male sex^15^, earlier age of onset of obesity^18^, higher baseline BMI, preoperative weight gain, wait time before surgery, hypertension and diabetes^2,17,18^. In addition, biomarkers associated with weight loss outcomes include alanine transaminase (ALT), aspartate transaminase (AST), white blood cell counts (WBC), insulin and hemoglobin A1c (HbA1c) levels^19^, preoperative triglyceride level^14^. Specific genotypes^15^ and race/ethnicity independent of health status and lifestyle behaviors^20^ have also been associated with postoperative weight loss. Preoperative mental status measured by pre-surgical cognitive function, personality, mental health, composite psychological variables, binge eating^21^, emotional food cravings^22^, history of physical abuse^23^, and egoism^24^ are also predictors. Some mental health diagnoses, including binge eating disorder^16^, can also predict long-term weight loss effects. However, these risk factors were identified from patients who received gastric band, Roux-en-Y gastric bypass, or one-anastomosis gastric bypass surgery.

Predictors for weight loss after LSG include gender, diabetes, preoperative obstructive sleep apnea (OSA), retired status^25^, the bougie size^26^, and the speed of contrast pass in the postoperative swallow study^27^. Patients with less than 12 months from surgery to steady-state, defined as the month when the patient has ≤ 3% EBWL, had a significantly lower weight loss at the 3- to 4-year follow-up compared to those with more than 12 months to steady-state^28^. Postoperative clinic non-attendance was also reported as a weight loss predictor. Missing at least 50% of postoperative visits was associated with less weight loss at 2-years^29^. Huang et al.^30^ also presented patients who had age less than 50 years old, preoperative alcohol consumption, without psychiatric history. Since many of these factors are not known before or immediately after surgery, it is difficult to advise caregivers when to implement more aggressive interventions to help patients achieve adequate weight loss.

Our scoring system uses a follow-up period as short as 1-month after LSG to more quickly identify patients with poor treatment response. For these patients, clinicians can intervene with more intensive clinic follow-ups, dietary education and evaluation, exercise programs, behavior modification, and support groups. Moreover, the scoring system can be used to assess EBWL associated with barriers to good habits and review the current treatment regimens to control depression, anxiety, and binge eating^31–34^. We believe these interventions offer better results in short-term weight loss as well as a higher chance of comorbidity remission, lower comorbidity recurrence, and lower risk of weight regain^4,5,35^.

There are weight loss predictor models generated from LSG. Cottam et al.^9^ established a prediction model based on comorbidities, including diabetes and/or sleep apnea, and the %EWL at 1-and 3-months after LSG to predict %EWL > 55% at 1 year. It had a 71% sensitivity, 91% specificity, 72% PPV and 91% NPV, but has not been validated. Van de Laaret al.^3^ presented bariatric weight loss charts with standard deviation and percentile curves based on weights measured after LSG from 3 large bariatric centers in the Netherlands. It aims to assess weight loss, weight-regain, and poor responders up to 7 years after sleeve gastrectomy and was validated by large studies (n > 500), reporting weight loss results after LSG with BMI > 35 kg/m^2^ and age ≥ 18 years with a minimum of 5-years follow-up. Although these are useful tools for predicting weight loss after LSG, they are not validated in Asian patients. Our results were generated from Asian populations, validated by Asian population and similarly showed that the weight loss in postoperative months 1 and 3 is important to identify patients who may be responding poorly to bariatric surgery. We believed the scoring system, 6M50LSG scoring system, can provides a reliable and objective evaluation to Asian patients who receive LSG.

There were limitations to our study. First, the retrospective, single-center non-randomized series design had a relatively small sample size, limiting the generalizability of our findings. Secondly, we excluded about half of the patients (150/334) due to incomplete data, which was not ideal for optimizing the model. To overcome this limitation, we separated the patients into two groups based on the surgeon to eliminate potential bias. Also, we included data from another hospital to strengthen the validity of our findings. Finally, we did not apply regular polysomnography to identify patients with OSA. However, we applied this scoring to patients with preoperatively diagnosed OSA and the model still had an acceptable ability to predict ≥ 50% EBWL after LSG.

## Conclusions

The 6M50LSG scoring system presented in this study provides a reliable and objective evaluation to closely monitor patients as early as 1- to 3-months post LSG, and apply more aggressive clinical follow-up, nutrition education, and lifestyle interventions to ensure an adequate EBWL in Asian population.
